# Rehabilitation Nutrition for Injury Recovery of Athletes: The Role of Macronutrient Intake

**DOI:** 10.3390/nu12082449

**Published:** 2020-08-14

**Authors:** Sousana K. Papadopoulou

**Affiliations:** Department of Nutritional Sciences and Dietetics, Faculty of Health Sciences, International Hellenic University, 57001 Thessaloniki, Greece; souzpapa@gmail.com; Tel.: +30-6944798916

**Keywords:** energy requirements, recovery, reduced muscle mass, sarcopenia, sport nutrition, injury

## Abstract

An adequate and balanced diet is of utmost importance in recovery and rehabilitation. “Rehabilitation nutrition” for injury recovery of athletes is similar to sports nutrition, except for the differences that concern the prevention of the risk or presence of sarcopenia, malnutrition, or dysphagia. Rehabilitation nutrition also aims, combined with training, to an adequate long-term nutritional status of the athlete and also in physical condition improvement, in terms of endurance and resistance. The aim of this paper is to define the proper nutrition for athletes in order to hasten their return to the sports after surgery or injury. Energy intake should be higher than the energy target in order to fight sarcopenia—that is 25–30 kcal/kg of body weight. Macro- and micro-nutrients play an important role in metabolism, energy production, hemoglobin synthesis, lean mass and bone mass maintenance, immunity, health, and protection against oxidative damage. Nutritional strategies, such as supplementation of suboptimal protein intake with leucine are feasible and effective in offsetting anabolic resistance. Thus, maintaining muscle mass, without gaining fat, becomes challenging for the injured athlete. A dietary strategy should be tailored to the athlete’s needs, considering amounts, frequency, type and, most of all, protein quality. During rehabilitation, simultaneous carbohydrates and protein intake can inhibit muscle breakdown and muscle atrophy. The long-term intake of omega-3 fatty acids enhances anabolic sensitivity to amino acids; thus, it may be beneficial to the injured athlete. Adequate intakes of macronutrients can play a major role supporting athletes’ anabolism.

## 1. Introduction

Sport injury and fear of injury are important barriers to participation in sport, despite the health benefits of sports activities. The incidence, prevalence and type of sport injuries vary from male to female as well as between age groups [1]. In general, the direct cost of an injury is determined by calculating the expense of using health-care services to avoid, diagnose, and treat injury and its complications [1]. A 52-week prospective analysis in elite adolescent athletes by Rosen et al. found that the prevalence of 1-year injury rate was 91.6%. The overall rate of injury was 4.1 for every 1000 h of sport exposure, and on average 3 out of 10 (30.8%) elite teenage athletes reported injuries per week [2]. A recent study by Polinder et al. estimated that the annual cost of sport injuries in patients visiting an emergency department in the Netherlands was 413 million euros every year [3]. There are 40 thousand football-related accidents in a year in Switzerland, which leads to a loss of 500 thousand working days. According to 2003 data from national insurance companies, the cost for the health system is about 19 million euros in the financial dimension [4]. Sport injuries cause health-related costs in excess of $1 billion dollars worldwide [4]. It is estimated that 3–5 million sports injuries occur in a year, according to data from the United Nations [4]. Football clubs suffer significant losses from success-related and financial factors when players, for whom huge sums of money have been paid, get injured [4].

Nutrition has a multi-dimensional effect on athletes’ physical and mental health and well-being [5,6,7,8]. An adequate and balanced diet is of utmost importance in recovery and rehabilitation. The human body requires energy and especially protein and unsaturated fatty acids to compensate for inflammation as well as a plethora of micronutrients, which contribute in healing.

Athletes should enjoy both nutritious and palatable food during rehabilitation phase. Therefore, their food selection should be in accordance to their personal habits and preferences, in addition to their needs.

Having a compromised post-surgical nutritional status may result in poor outcomes, including complications, infections, and long hospital stay; hence, the aim of an optimized diet is to maximize the response of injury/surgical treatment, to maintain the recommended body weight and body fat status according to their sport and to ensure fast return to their training schedule and performance ability.

## 2. Rehabilitation Nutrition for Injury Recovery of Athletes

Rehabilitation nutrition is used in International Classification of Functioning, Disability, and Health guidelines combining both rehabilitation and nutrition, in order to improve mental and physical function facilitating the daily activities [9]. During rehabilitation, the primary goal is to achieve the fastest healing and to return to competition, using the most effective resources, where nutrition is an important component. After injury or surgery, it is the critical time to improve athletes’ eating attitudes and behaviors, which lead towards the healing process and/or enhance performance afterwards [10]. According to Wakabayashi and Sakuma [9] “rehabilitation nutrition” is similar to sports nutrition. Of course, the term also includes the assessment of the risk or presence of sarcopenia, malnutrition, or dysphagia. For the purposes of this review, there is an extensive overview of specific nutritional intervention technics, especially focused on the macronutrients needed in the rehabilitation phase of an athlete that has suffered injury or/and surgery. In such cases nutritional intervention also aims, combined with training, to an adequate long-term nutritional status of the athlete and also in physical condition improvement, in terms of endurance and resistance.

## 3. Energy Requirements

Energy balance holds a prominent position in sustaining muscle mass, in order for amino acids not to be oxidized, but to be used for muscle protein synthesis (MPS) [11]. In general, an injured athlete decreases his/her physical activity and, thus, his/her energy requirements. Adequate energy intake should be the first nutritional consideration as negative energy balance accelerates muscle loss especially in disuse/immobility period [12]. Additionally, an overall reduction in energy intake also leads to a decline in dietary protein, to an amount that it fails to meet the recommended intake for muscle mass maintenance in athletes [13]. Energy intake should be higher than the energy target in order to fight sarcopenia—that is 25–30 kcal/kg of body weight [14,15]. However, providing excess energy does not further attenuate muscle loss, but rather results in an increased fat deposition [16].

Resistance training and prolonged exercise per se are anabolic, as they provide the most powerful anabolic stimulus to MPS [17]. In conditions of energy restriction or sudden inactivity, as a result of surgery or injury, elevating protein intakes to 2.0 g/kg/day or even higher [18,19] may be advantageous in preventing fat free mass (FFM) loss [17].

Adequate food and fluid intake are vital to ensure athletic performance and recovery. Macro- and micro-nutrients play an important role in metabolism, energy production, hemoglobin synthesis, lean mass and bone mass maintenance, immunity, health, and protection against oxidative damage [20].

## 4. Induced Muscle Atrophy

Injuries and surgeries require a long period of immobilization, involving avoidance of muscle contraction or weight bearing activities of the affected muscles during the initial phase (about 6 weeks). This leads to muscle disuse atrophy at a rate of 0.5% per day, accompanied by 50% decline in MPS [21] and muscle loss that can be equal to 150–400 g of muscle tissue from a leg within the early stages (1–2 weeks) [22]. During this period rapid muscle strength loss also occurs. Thus, an 8% quadriceps’ muscle loss can induce a 23% decline in muscle strength [13]. The extent of muscle atrophy influences the duration of required rehabilitation [19]. Muscle atrophy etiology is multifactorial and can be partly attributed to acute inflammatory and hormonal response. Elevated potent atrophy-inducing cytokines may contribute to the delayed strength and functional recovery [23]. In addition, during misuse, bone calcium loss occurs [24], metabolic rate is decreased [25], insulin sensitivity declines, whilst fat deposition increases [26].

Protein synthesis depression is primarily due to muscle disuse atrophy whilst proteolysis may not be enhanced. Decline in MPS rate in post-absorptive and postprandial states induce the development of a phenomenon called “anabolic resistance” to food intake i.e., a decline in postprandial protein synthesis’ response [21]. Thus, anabolic resistance can be partially responsible for muscle disuse and muscle atrophy. Apart from anabolic resistance, inactivity can also lead to systemic inflammation, insulin resistance, reduction in activation, recruitment of satellite cells and decrease in capillary density, resulting in loss of muscle mass and strength [14,27].

However, it should be noted that the inflammation factor that accompanies the injury or surgery, plays a crucial role in muscle wasting in ways that have not yet been clarified to the fullest extend due to lack of satisfactory evidence that could be attributed to practical limitations such as heterogeneity in pathology- and disease-related complications [28]. There is definitely a need for longitudinal evidence in which the changes in systemic and muscle inflammation are connected to changes in muscle molecular signaling (anabolic, catabolic, oxidative stress, Advanced Glycation End Products or AGEs, etc.), muscle mass, and muscle strength [28]. For that reason, it should be clarified that some of the evidence that refer to young healthy adults and young athletes can be considered representative and useful but do not necessarily take into account the way an injury, surgery, or the inflammatory response would affect the mechanism that is involved in induced muscle atrophy. Nutritional strategies, such as supplementation of suboptimal protein intake with leucine are feasible and effective in offsetting anabolic resistance.

## 5. Macronutrient Requirements

### 5.1. The Key Role of Protein

Compensating anabolic resistance and aiming at optimizing the anabolic response to protein consumption in order to preserve muscle mass, and avoid body fat gain are nutritional challenges [13]. A significant amount of evidence indicates that milk-based protein after resistance exercise acts favorably on body composition and muscle strength [29,30]. A higher protein intake is related to muscle mass retention. Additionally, it has been noted that excess protein is metabolically difficult to be turned into fat, when there is no excess energy intake.

Type, amount, timing, and frequency of protein intake are of key importance in restricting strength and muscle mass loss during recovery [13]. Whey protein extracts have contiguous amino acid content and in approximate proportions with that found in skeletal muscles [31,32]. According to Reidy and Rasmussen [33], protein quantity seems to be more important than the type of protein, as no significant differences were found in muscle mass or strength under resistance training, when the threshold of 2 g of leucine was reached—an amount that can be achieved with 20–30 g of high-quality protein. It is of paramount importance that in energy deficit and in health disturbances like ageing, inflammation and sickness a higher dose of protein is advised to maximize muscle protein synthesis, whereas a lower dose is efficient for well-trained athletes [33].

Considering the 2 to 4 h accretion window of protein, allowed by each meal consumed, the feeding strategy is also an important factor that affects muscle synthesis. During the long period of the day (12 h) through post-prandial protein accretion, the maximal protein anabolism could be achieved through the increase of meal frequency combined with an adequate and evenly spaced offer of protein amount across all meals [34]. Mamerow et al. [35] found that 24 h muscle synthesis rate was 25% more effective in healthy women and men when fed with protein evenly distributed across breakfast, lunch, and dinner compared to isoenergetic and isonitrogenous diets with uneven protein distribution across meals. According to this, it is important to avoid low protein breakfast/snack or dinner/high protein lunch, to maximize protein accretion [13]. Thus, protein intakes of 1.3–1.8 g/kg body weight when spaced over as 3–4 isonitrogenous meals induce maximized muscle synthesis [17,36]. The recommendation of Wall et al. [19] is even higher, which is 1.6–2.5 g/kg, evenly spread across the day, 4–6 times, every 3–4 h, in amounts of 20–35 g, which contain high amounts of leucine (2.5–3 g). Similarly, Moore et al. [34] came up to the above suggestion of 4 to 6 meals high in protein for athletes during resistant training in order to maximize muscle strength and muscle mass. Areta et al. [37] reported that 20 g of whey protein consumed every 3 h was superior to either more frequent (every 1.5 h) or less frequent (every 6 h) feeding patterns for stimulating MPS throughout the day.

The overall metabolism, both in the short- and the long-term, muscle recovery rate, and MPS could be improved from the consumption 2 h after dinner and 30 min before sleep of protein-rich drinks. Furthermore, it is indicated that 30–40 g ingestion of casein protein 30 min before sleep or via nasogastric tube benefit overnight MPS in both young and older men, respectively [38]. Tang et al. [39] concluded that a significant increase on muscle protein synthesis rate while at rest and in response to resistance exercise can be induced due to the consumption of whey, soy, and casein protein sources. However, Burd et al. [40] found that the consumption of 20 g of isolated whey protein leads to greater rates of MPS than casein in micellar form both at rest and after resistance exercise in healthy elderly men.

It has been found that in order to increase amino acid availability during overnight sleep, protein ingestion is advised prior to sleep, as it is sufficiently digested and absorbed [41]. Sufficient amino acid availability during the hours of sleep is important for muscle recovery as it has been proved to stimulate muscle protein synthesis rates and benefit whole-body protein net balance [41]. More specifically, to induce a robust and sufficient stimulation of muscle protein during overnight recovery, a minimum amount of 40 g of dietary protein is advised [41].

Both high-quality protein from the consumption of whole foods and dietary supplements of isolated proteins (whey, casein, egg, meat and soy) are beneficial to the accretion of postprandial protein and induce MPS [42]. However, whey protein seems to stimulate postprandial muscle protein accretion in a more extensive rate in comparison to the way that casein and casein hydrolysate seem to affect older men. This effect is attributed to a combination of whey’s faster digestion and absorption kinetics and higher leucine content [40]. Probably, the ability of whey protein to retain muscle mass and promote fat loss can be related to its high content in essential and Branched-Chain Amino Acids (BCAAs) and particularly leucine. Leucine can induce a reduction of urinary nitrogen loss and has been shown to affect the anabolic kinetics of muscle protein after 36 h of fasting, and LWH (leucine-rich whey protein beverage) distinctly activates the mTOR (mammalian target of rapamycin) pathway [43]. However, further research is needed [44]. A later study of the above researchers confirmed that BCAA and mainly leucine played a prominent role in stimulating MPS [17]. The greater effect of whey, in comparison to casein and casein hydrolysate, to the postprandial muscle protein accretion was attributed to its quicker digestion, the absorption mechanism kinetics and its amino acid composition and especially its content in leucine [42]. However, two studies from the same research group concluded that, in young and elder individuals, the consumption of casein which is considered to be a slowly digested protein, prior or during sleep (intragastrically) [45], could affect beneficially MPS nocturnally through hyperaminoacidemia with the following positive results in protein balance throughout post-exercise overnight recovery.

In 80 kg resistance-trained young men, the advised dose of whey protein in order to achieve the optimal stimulation of MPS in rested and exercised muscle is 20 g. Higher doses stimulate urea genesis and oxidation of amino acids and do not result in further myofibrillar MPS stimulation [46]. In older non-frail adults, myofibrillar MPS increase occurred with 20 g of whey ingestion at rest. However, older resistance exercised muscles responded with increased MPS at all protein doses but to a greater extend with 40 g of protein [47]. Likewise, 20 g of high-quality protein, which is considered to be equivalent to 20 g essential amino acids (EAAs) is related to higher rates of MPS in older muscle [16].

Thus, we could speculate that the same suggestion is valid for the injured athlete. In order to overcome the disuse-induced anabolic resistance to protein intake, that is for example a reduced responsiveness to protein ingestion, injured athletes also require 35–40 g of protein intake to increase postprandial MPS [19].

Considering the above, during recovery from injury, maintaining adequate protein intake, while manipulating the amount and the type of protein but also the difference between the rapidly in contrast to the more slowly digested proteins in the nutritional plan are of crucial importance. However, the co-ingestion of other macro and micronutrient is also a prerequisite and will be further discussed below.

### 5.2. Carbohydrates (CHO)

The level of endogenous CHO availability is known to affect protein synthesis and skeletal muscles during exercise. Low levels of CHO influence protein degradation, increase muscle protein breakdown, and reduce net protein balance, especially in conditions of low muscle glycogen compared with high-glycogen availability [48].

The underlying mechanism of CHO for metabolic responses is unclear and findings are controversial within different exercise or recovery settings. Thus, the role of CHO in the recovery phase needs further investigation for clear conclusions. Despite limited evidence on CHO or on fatty acids’ benefits compared with protein intake [49], a well-balanced diet that includes adequate amounts of proteins, CHO, antioxidants, and moderate fat is recommended for the rehabilitation of the injured athlete.

### 5.3. Fat

According to recent data, fish oil derived omega-3 fatty acid supplementation may be beneficial to the injured athlete, having anti-inflammatory properties [50]. Among healthy individuals of all ages [51,52] long-chain supplementation with omega-3 fatty acids at dosage 4 g/day increased anabolic sensitivity to amino acids and the response of muscle protein synthesis to hyperinsulinemia-hyperaminoacidemia, resulting in augmented muscle cell size. Particularly, it was unlikely that the positive effect of omega-3 fatty acids on muscle anabolic action was associated to their anti-inflammatory properties; rather, there was an apparent increase in the sensitizing effect on the molecular signaling regulating muscle protein synthesis. It should be noted that above studies’ subjects were young and older healthy adults who had no inflammation or other related health conditions at baseline. Deficiencies of the fat-soluble vitamins D and K increase the risk of development and progression of osteoarthritis [53,54]. Musumeci et al., highlighted that the consumption of Mediterranean diet and extra virgin olive oil can help to attenuate and overcome inflammation after injury in the articular cartilage, preventing osteoarthritis [55].

### 5.4. Supplements with Macronutrients

#### 5.4.1. Leucine and Hydroxy-methylbutyrate (HMB)

Leucine has been long suggested to have a potential role as a regulator of protein metabolism [56]. For the treatment of injury, the supplementation of additional nutrients, such as BCAAs (including leucine), creatine, omega-3 fatty acids and HMB have been proposed as beneficial on MPS [19].

According to a recent systematic review, a high (>200 mg/kg/day) and long-term (>10 days) daily supplementation of BCAA (including leucine, valine) could contribute in outcomes of low to moderate exercise-induced muscle damage, especially if consumed prior to the damaging exercise in healthy subjects [57]. Findings in elderly people showed that it is important to preserve a high proportion of leucine in an essential amino acid mixture, in order to reverse the suboptimal muscle protein synthesis—though this did not apply to younger subjects [58]. In another study, the synthesis of the skeletal muscle of aged mice was seen to benefit from the administration of leucine-enriched whey protein supplements, although this effect was not observed in the case of isolated leucine [59]. In fact, among elderly men, the anabolic rate has been found to increase due to the co-ingestion of 2.5 g of crystalline leucine with pure dietary protein [60]. The inhibitory effect of leucine on catabolism also applies to the concept of leucine-enriched meals intake during recovery of the injured athlete, mainly during the early stages, when catabolic processes are usually intense [19].

Of interest is leucine’s preferential transamination to produce HMB [19]. The recommended dosage is 3 g/day. The use of supplements can enhance the adaptive response to exercise as it can slow down the rate of protein breakdown. On the other hand it promotes protein synthesis, cholesterol synthesis, secretion of growth hormone and IGF-I (Growth hormone and Insulin-like growth factor-I) mRNA synthesis. All this factors lead to the increase of the proliferation and differentiation of satellite cells and inhibiting apoptosis. It is stated that the positive effects on strength and fat-free mass, due to the consumption of HMB are insignificant. Furthermore, the effects on muscle damage have not yet been well defined. All recent reports that state an increase of strength similar to the effects of steroid consumption or beneficial results on FFM, as well as inhibitions of the rates of muscle damage due to HMB-free acid supplementation, have not been reproduced and seem unlikely to be accurate [61,62].

In a study with young men, HMB could stimulate acute muscle protein strength and could reduce muscle protein breakdown, presenting similar effects with leucine [63]. In healthy older subjects, supplementation with HMB, during 10 days of bed rest, could preserve muscle mass [64].

Regarding the results of the use of HMB, the only collected data are related to experiments conducted after periods of extreme inactivity or muscle disuse or during recovery from injury, in older adults and after a 10-day bed rest. Achieving the advised protein intake recommendations from normal dietary protein or whole protein supplements could most likely assure the optimal results in the muscle recovery rate, hence HMB supplements may not result in further positive results [61]. However, according to Jakubowski et al., who performed a systematic review and meta-analysis on the accumulated data regarding HMB supplementation, there were no significant effects for FFM, fat mass (FM), nor did it result to increase of strength [65]. As a result, more research is needed about such beneficial effects of HMB, especially alongside a high leucine diet, to prove its value to the injured athlete.

#### 5.4.2. Lysine and Pipecolic Acid

Lysine, one of the essential amino acids, is also found in great quantities in muscles, and is associated with muscle mass [66]. In a metabolomic analysis, it was found that serum pipecolic acid, a lysine metabolite, was positively associated with muscle mass and muscle strength, but negatively associated with age in Caucasian women. In mice, lysine administration could suppress proteolysis in skeletal muscle in fasted state [67] while continuous feeding of a high-lysine diet could increase muscle mass [68]. Lysine supplementation in rats could reduce the increased skeletal muscle autophagy, caused by a low protein diet and activate Akt (protein kinase B)/mTOR signaling pathways. Pipecolic acid could also stimulate protein synthesis rates, and contribute to the reduction of muscle protein degradation [69].

#### 5.4.3. Omega-3 Fatty Acids

Unsaturated fatty acids act as antioxidants improving lipid oxidation [70]. The highest antioxidant phenolic content is found in virgin olive oil (VOO) compared with other ordinary olive oils [71].

In healthy individuals of all ages, fish oil derived omega-3 fatty acids regulate muscle protein synthesis [49]. In particular, the long-term intake of omega-3 fatty acids (4 g/day) enhances anabolic sensitivity to amino acids [51,52], having a sensitizing effect on the molecular signaling pathways. These findings suggest that fish oil derived omega-3 fatty acid intake may also be beneficial to the injured athlete—although relevant data are few and less consistent compared with protein intake [49].

In the context of sport-related injuries, omega-3 fatty acid supplementation is proposed for anti-inflammatory effects, as well as immuno-modulatory properties [70,72]. Such properties have also been assessed for injury prevention. Qualities of omega-3 fatty acids are related to positive impacts against inflammation and disturbance of the immune system, while low intake of fat may increase injuries or the seriousness of injury, by strengthening inflammatory response [73].

Evidence from rats, however, is not in line with the above expectations regarding all types of injury healing. In rats, supplementation of different types of edible oils delayed cutaneous wound healing, negatively

Affecting collagen regeneration [74], while other studies indicated that high fish oil consumption could inhibit wound healing and muscle mass recovery [70]. Open wounds need even more collagen and new growth of the capillaries. This cycle relies on the favorable nutritional status of the patient to produce healthy tissue of granulation [75]. Regarding immobilization or reduced activity after injury, studies showed that rats with high fish oil diets experienced less muscle loss during hind limb immobilization [76], but similar diets on rodents had a restraining influence on recovery after hind limb suspension [73]. On the contrary, omega-3 supplementation at a dosage of 2 g/day for 15 days benefited the modulation of oxidative biomarkers during rehabilitation of patients who had undergone knee surgery [77].

According to the above findings, fish oil intake probably ameliorates muscle loss during immobilization, but it does not seem to be effective for muscle gain. Therefore, suggestions for intake of fish oil in injured people should consider the appropriate dose, which has not yet been established [73]. Further research is needed to provide evidence-based results about the efficacy of omega-3 fatty acid and/or creatine supplementation on muscle loss or anabolic resistance in injured athletes.

## 6. Summing up the Evidence

The role of the dietitian is crucial, so as to plan an individualized nutrition program, which takes into consideration the athlete’s needs and preferences, and according to the current guidelines and recommendations, regarding nutrition during recovery.

Several strategies applied during rehabilitation phase include the nutritional factor, in order to enhance the physiological response and hasten the return to the play. The procedures can be demanding, particularly during the early stages of muscle disuse, while the extent of muscle loss strongly affects the duration of this phase. Thus, maintaining muscle mass, without gaining fat, becomes challenging for the injured athlete.

Of crucial importance is the adequate protein intake, as it is associated with muscle mass retention and strength, while it can protect from fat deposition. A dietary strategy should be tailored to the athlete’s needs, considering amounts, frequency, type and, most of all, protein quality. Similar strategies can also be applied to the injured athlete for muscle synthesis stimulation. Recommendations on protein supplementation, however, should be conservative, following an overall high-quality dietary intake.

CHO availability influences protein synthesis and skeletal muscle. CHO intake facilitates protein accretion and improves recovery. During rehabilitation, simultaneous CHO and protein intake can inhibit muscle breakdown and muscle atrophy, although research findings regarding CHO intake are controversial within different exercise or rehabilitation settings, compared with findings on protein intake.

Evidence on the benefits of fatty acids is inconsistent. Fatty acids and fish oil are recognized as antioxidants, having anti-inflammatory properties. The long-term intake of omega-3 fatty acids enhances anabolic sensitivity to amino acids; thus, it may be beneficial to the injured athlete. Intake of fatty acids, however, should follow a well-balanced diet, also including CHO, proteins, and other micronutrients, considering appropriate doses and other factors of the injured human. A detailed summary of all the data reviewed is presented on Table 1. 

Other supplements suggested for muscle damage treatment and protein synthesis include branched chain amino acids, leucine, and HMB. Leucine-enriched meals are recommended during recovery, especially on the early stages. More research is needed, however, on the potential role of HMB to the injured athlete, alongside a high leucine diet.

## 7. Conclusions

Conclusively, both during exercise and rehabilitation, adequate intakes of macronutrients can play a major role supporting athletes’ anabolism. Future research is warranted to clarify the underlying mechanisms of nutrients, especially regarding injury treatment, as their efficacy has not yet been assessed satisfactorily. Dietary protocols should consider doses, timing, rehabilitation time, type and quality of nutrients, as well as the type of injury, and the injured body part. Monitored evaluation should follow, in order to assess nutrient indicators and to avoid levels above sufficiency. High-quality nutrient-rich mixed diets are suggested. Biomedical indices and vitamin and mineral levels should be assessed and monitored, in order to avoid unnecessary supplementation.

## Figures and Tables

**Table 1:** Primary studies on the role of macronutrients on rehabilitation.

First Author	Nutrient	Design	Results
Josse et al., 2011 [29]	Protein	90 healthy, premenopausal, overweight, and obese women were randomized to 3 groups (*n* = 30/group): high protein, high dairy, adequate protein, medium dairy, and adequate protein, low dairy hypocaloric diets.	Diet- and exercise-induced weight loss with higher protein and increased dairy product intakes promotes more favorable body composition changes in women characterized by greater total and visceral fat loss and lean mass gain.
Hartman et al., 2007 [30]	Protein	56 healthy young men who trained 5 days/week for 12 weeks randomly assigned to consume drinks immediately and again 1 h after exercise: fat-free milk. (Milk; *n* = 18); fat-free soy protein (Soy; *n* = 19) that was isoenergetic, isonitrogenous, and macronutrient ratio matched to Milk; or maltodextrin that was isoenergetic with Milk and Soy (control group; *n* = 19).	Post-exercise consumption of milk promotes greater hypertrophy during the early stages of resistance training in novice weightlifters when compared with isoenergetic soy or carbohydrate consumption.
Areta et al., 2013 [37]	Protein	24 healthy trained males were assigned to three groups of 8 and undertook a bout of resistance exercise followed by ingestion of 80 g of whey protein throughout 12 h recovery in one of the following protocols: 8 × 10 g every 1.5 h, 4 × 20 g every 3 h; or 2 × 40 g every 6 h.	20 g of whey protein consumed every 3 h was superior to either more frequent (every 1.5 h) or less frequent (every 6 h) feeding patterns for stimulating MPS (muscle protein synthesis) throughout the day.
Moore et al., 2008 [34]	Protein	6 healthy young men undertook 5 intense leg-based resistance exercise programs. After exercise, participants consumed, in a randomized order, drinks containing 0, 5, 10, 20, or 40 g, whole egg protein.	Ingestion of 20 g intact protein is sufficient to maximally stimulate muscle and albumin protein synthesis after resistance exercise, which corresponds to ~8–9 g essential amino acids (EAAs) and ~1.8 g leucine. 4 to 6 meals high in protein for athletes during resistant training to maximize muscle strength and muscle mass. BCAA (Branched-chain amino acid) and mainly leucine play a prominent role in stimulating MPS during resistant training to maximize muscle strength and muscle mass.
Mamerow et al., 2014 [35]	Protein	7-day crossover feeding design study with a 30-day washout period, to examine changes in muscle protein synthesis in response to isoenergetic and isonitrogenous diets with protein at breakfast, lunch, and dinner distributed evenly or skewed on 8 participants.	24 h muscle synthesis rate was 25% more effective in healthy women and men when fed with protein evenly distributed across breakfast, lunch and dinner compared to isoenergetic and isonitrogenous diets with uneven protein distribution across meals.
Tang et al., 2009 [39]	Protein	3 groups of 6 healthy young men performed a bout of unilateral leg resistance exercise followed by the consumption of a drink containing an equivalent content of essential amino acids (10 g) as either whey hydrolysate, micellar casein, or soy protein isolate.	All protein sources significantly increased muscle protein synthesis rates both at rest and in response to resistance exercise.
Burd et al., 2012 [40]	Protein	Healthy elderly men were divided into two groups of 7 & performed unilateral leg resistance exercise followed by the consumption of isonitrogenous quantities (20 g) of casein or whey.	Ingestion of isolated whey protein supports greater rates of MPS than micellar casein both at rest and after resistance exercise in healthy elderly men.
Pennings et al., 2011 [42]	Protein	48 older men were randomly assigned to ingest a meal-like amount (20 g) of intrinsically l-[1-(13)C]phenylalanine-labeled whey, casein, or casein hydrolysate. Protein ingestion was combined with continuous intravenous l-[ring-(2)H(5)]phenylalanine infusion to assess in vivo digestion and absorption kinetics of dietary protein.	Whey protein is more effective on postprandial muscle protein accretion than casein and casein hydrolysate in older mend to whey’s faster digestion and absorption kinetics and higher leucine content.
Rittig et al., 2017 [43]	Protein	Randomized crossover trial to compare a specific leucine-rich whey protein beverage (LWH) with isocaloric CHO (carbohydrates), soy protein (SOY), and soy protein +3 g HMB (hydroxymethylbutyrate) during fasting-induced catabolic conditions	LWH and HMB have superior anabolic effects on muscle protein kinetics after 36 h of fasting, and LWH distinctly activates the mTOR pathway. These novel findings suggest that leucine-rich whey protein and/or HMB are specifically beneficial during fasting-induced catabolic conditions.
Res et al., 2012 [45]	Protein	16 healthy young males performed a single bout of resistance-type exercise in the evening after a full day of dietary standardization followed by recovery nutrition (20 g of protein, 60 g of CHO) immediately after exercise. Thereafter, 30 min before sleep (2330 h), subjects ingested a beverage with protein or placebo.	Protein ingested immediately before sleep is effectively digested and absorbed, thereby stimulating muscle protein synthesis and improving whole-body protein balance during post exercise overnight recovery.
Witard et al., 2013 [46]	Protein	48 volunteers consumed a standardized, high-protein (0.54 g/kg body mass) breakfast. 3 h later, they underwent a bout of unilateral exercise and ingested 0, 10, 20, or 40 g whey protein isolate immediately (∼10 min) after exercise.	A 20-g dose of whey protein is sufficient for the maximal stimulation of post-absorptive rates of myofibrillar MPS in rested and exercised muscle of ~80-kg resistance-trained, young men. A dose of whey protein >20 g stimulates amino acid oxidation and ureagenesis.
Yang et al., 2012 [47]	Protein	37 elderly men completed a bout of unilateral leg-based resistance exercise before ingesting 0, 10, 20 or 40 g of whey protein isolate.	Resistance exercise increases MPS in the elderly at all protein doses, but to a greater extent with 40 g of whey ingestion, in contrast to younger adults, in whom post-exercise rates of MPS are saturated with 20 g of protein, exercised muscles of older adults respond to higher protein doses.
Katsanos et al., 2005 [58]	Leucine	2 elderly (*n* = 10 & 10) and 2 young (*n* = 8 & 8) groups were studied before and after ingestion of 6.7 g of EAAs with different %leucine.	increasing the proportion of leucine in a mixture of EAA can reverse an attenuated response of muscle protein synthesis in elderly but does not result in further stimulation of muscle protein synthesis in young subjects.
Dijk et al., 2018 [59]	Leucine	Overnight fasted C57/BL6RJ mice at 25-mo of age received an oral gavage with leucine or whey-protein enriched with leucine (0.75 g/kg bodyweight total leucine in both) or 0.5 mL water (fasted control).	MPS is stimulated in aged mice by leucine-enriched whey protein but not by leucine administration only.
Wall et al., 2013 [60]	Leucine	24 elderly men randomly assigned to ingest 20 g intrinsically L-[1-(13)C]phenylalanine-labeled casein protein with (PRO+LEU) or without (PRO) 2.5 g crystalline leucine.	Leucine co-ingestion with a bolus of pure dietary protein further stimulates post-prandial muscle protein synthesis rates in elderly men.
Wilkinson et al., 2013 [63]	Leucine and HMB	15 young healthy men, who were recreationally active but not involved in a formal training program, were randomized to receive 3.42 g HMB or 3.42 g Leucine.	HMB and Leucine ↑ anabolic signaling but it was more pronounced with Leucine. HMB consumption also attenuated muscle protein breakdown in an insulin-independent manner.
Deutz et al., 2013 [64]	HMB	Randomized, controlled, double-blinded, parallel-group design study was carried out in 24 healthy (SPPB ≥ 9) older adult subjects (20 women, 4 men), confined to complete bed rest for ten days, followed by resistance training rehabilitation for eight weeks. The experimental group received HMB (calcium salt, 1.5 g twice daily—total 3 g/day) and the control group an inactive placebo powder.	HMB supplementation preserves muscle mass during 10 days of bed rest.
Zhao et al., 2018 [66]	pipecolic acid	Analysis of metabolomic profiles of 136 Caucasian women who exercised 3 times per week.	Pipecolic acid was positively associated with muscle mass and muscle strength, but negatively associated with age.
Sato et al., 2013 [67]	Lysine	Fasted rats were administered 22.8–570 mg Lys/100 g body weight and the rates of myofibrillar protein degradation were assessed for 0–6 h after Lys administration.	Lys is able to suppress myofibrillar protein degradation at least partially through the autophagic-lysosomal pathway, not the ubiquitin-proteasomal pathway, whereas Lys might be unable to stimulate protein synthesis within this time frame.
Sato et al., 2016 [69]	Lysine & metabolites	Exploration of the effect of Lys metabolites, L-2-aminoadipic acid (2-AA) and L-pipecolic acid (Pip), on protein turnover in C2C12 myotubes.	In C2C12 myotubes, L-2-aminoadipic acid could suppress autophagy and pipecolic acid could stimulate the rates of protein synthesis, and these metabolites may contribute to exert effect of Lysine on protein turnover.
Howarth et al., 2010 [48]	Carbohydrates	6 men cycled at approximately 75% peak O_2_ uptake (Vo(2peak)) to exhaustion to reduce body CHO stores and then consumed either a high-CHO or low-CHO diet for 2 days before the trial in random order.	The whole-body net protein balance was reduced in the L-CHO group, largely due to a decrease in whole body protein synthesis.
Smith et al., 2011 [51]	Fat	9 25–45-year-old healthy subjects supplemented for 8 weeks with 4 g of Lovaza^®^/day.	Basal muscle protein fractional synthesis rate and basal signaling element phosphorylation remained unchanged after LCn-3PUFA supplementation, but the anabolic response to insulin and amino acid infusion was greater. Muscle protein concentration and the protein/DNA ratio were both greater after supplementation.
Smith et al., 2010 [52]	Fat	16 healthy, older adults were randomized to receive either omega-3 fatty acids or corn oil for 8 weeks.	Omega-3 fatty acid supplementation had no effect on the basal rate of muscle protein synthesis, but augmented the hyperaminoacidemia-hyperinsulinemia-induced increase in the rate of muscle protein synthesis, accompanied by greater increases in p70s6k(Thr389) phosphorylation.
Musumeci et al., 2013 [55]	Fat	Morphological Study on experimental bones.	Inflammation process causes stress to chondrocytes that will die as a biological defense mechanism, and will also increase the survival of new chondrocytes for maintaining cell homeostasis. Healthy diet plays important anti-inflammatory role.
Fito et al., 2007 [71]	Fat	Randomized Controlled Trial with 372 subjects at high cardiovascular risk who were recruited into PREDIMED trial.	Individuals at high cardiovascular risk who improved their diet toward a TMD pattern showed significant reductions in cellular lipid levels and LDL oxidation.
Otranto et al., 2010 [74]	Omega-3 fatty acids	30 days before wounding, rats were started on daily supplements of sunflower oil, linseed oil, fish oil, or water. On day 0, an excisional wound was made on the back of each animal and euthanasia was undertaken on day 14.	Different types of edible oils delayed cutaneous wound healing, negatively affecting collagen regeneration.
You et al., 2010 [76]	Omega-3 fatty acids	Rats were fed a corn-oil- (control) or fish-oil-based diet for 2 weeks, and then subjected to 10 days hindlimb immobilization while still receiving the same diets.	Rats with high fish oil diets experienced less muscle loss during hind limb immobilization.
Vidmar et al., 2016 [77]	Omega-3 fatty acids	Prospective, randomized, controlled, and single blinded clinical trial, with 25 patients who underwent anterior cruciate ligament reconstruction, randomly assigned to 2 g of omega-3 for 15 days after surgery and control (no supplementation).	Omega-3 supplementation benefited the modulation of oxidative biomarkers during rehabilitation of patients who had undergone knee surgery.
Shaw et al., 2019 [75]	Leucine, omega-3 fatty acids	Comparison of rehabilitation program undertaken by two professional rugby athletes’ anterior cruciate ligament (ACL), with the addition of an evidence-based supplementation (gelatine and vitamin C) and exercise protocol focused on collagenous tissue. Players undertook a structured rehabilitation program for 34 weeks before being clinically assessed ready to play.	Players saw minimal changes in body composition in the early rehabilitation period (P1—0.8 kg; P2—0.4 kg). Leg lean mass reduced in both legs of Player 1 (Injured—0.8 kg, Non-injured—0.6 kg) at 17 weeks, with Player 2 only experiencing a loss of 0.3 kg of lean tissue in the injured leg. Both players returned to baseline body compositions after 24 weeks. Leg strength returned to a maximum at 24 and 15 weeks, respectively, with knee function returning to baseline by 30 weeks.
